# Erratum to “Evaluation of the Expression of C-Kit (CD117) in Ependymomas and Oligodendrogliomas”

**DOI:** 10.1155/2014/180132

**Published:** 2014-01-30

**Authors:** Lisiane Silveira Zavalhia, Mirian Romitti, Gabriel Corteze Netto, Giovana Tavares dos Santos, Rosalva Thereza Meurer, Arlete Hilbig, Mariana Bohns Michalowski, Ligia Maria Barbosa Coutinho, Marlise de Castro Ribeiro

**Affiliations:** ^1^Programa de Pós Graduação em Patologia da Universidade Federal de Ciências da Saúde de Porto Alegre (UFCSPA), 90050-170 Porto Alegre, Brazil; ^2^Programa de Pós Graduação em Ciências Médicas da Universidade Federal do Rio Grande do Sul (UFRGS), 90040-060 Porto Alegre, Brazil; ^3^Laboratório de Pesquisa em Patologia da Universidade Federal de Ciências da Saúde de Porto Alegre (UFCSPA), 90050-170 Porto Alegre, Brazil; ^4^Departamento de Clínica Médica da Irmandade Santa Casa de Misericórdia de Porto Alegre, Porto Alegre, 90020-090 Porto Alegre, Brazil; ^5^Departamento de Pediatria da Irmandade Santa Casa de Misericórdia de Porto Alegre, Porto Alegre, 90020-090 Porto Alegre, Brazil

We came through this erratum and declare that we are aware that the author Ligia Maria Barbosa Coutinho, Professor at the Graduate Program in Pathology of Universidade Federal de Ciências da Saúde de Porto Alegre (UFCSPA), Brazil, was a part of the implementation of this paper, and, by our mistake, was absent from the list of authors. We would like to add Ligia Maria Barbosa Coutinho as a coauthor.

